# Development of copy number assays for detection and surveillance of piperaquine resistance associated *plasmepsin 2/3* copy number variation in *Plasmodium falciparum*

**DOI:** 10.1186/s12936-020-03249-x

**Published:** 2020-05-13

**Authors:** Megan R. Ansbro, Christopher G. Jacob, Roberto Amato, Mihir Kekre, Chanaki Amaratunga, Sokunthea Sreng, Seila Suon, Olivo Miotto, Rick M. Fairhurst, Thomas E. Wellems, Dominic P. Kwiatkowski

**Affiliations:** 1 Wellcome Sanger Institute, Hinxton, UK; 2 Laboratory of Malaria and Vector Research, National Institute of Allergy and Infectious Diseases, National Institutes of Health, Rockville, MD USA; 3 Mahidol-Oxford Tropical Medicine Research Unit, Mahidol University, Bangkok, Thailand; 4 National Center for Parasitology, Entomology, and Malaria Control, Phnom Penh, Cambodia; 5 Centre for Genomics and Global Health, Wellcome Centre for Human Genetics, Oxford, UK

**Keywords:** Malaria, Piperaquine, Plasmepsin, qPCR, Cambodia, Copy number

## Abstract

**Background:**

Long regarded as an epicenter of drug-resistant malaria, Southeast Asia continues to provide new challenges to the control of *Plasmodium falciparum* malaria. Recently, resistance to the artemisinin combination therapy partner drug piperaquine has been observed in multiple locations across Southeast Asia. Genetic studies have identified single nucleotide polymorphisms as well as copy number variations in the *plasmepsin 2* and *plasmepsin 3* genes, which encode haemoglobin-degrading proteases that associate with clinical and in vitro piperaquine resistance.

**Results:**

To accurately and quickly determine the presence of copy number variations in the *plasmepsin 2/3* genes in field isolates, this study developed a quantitative PCR assay using TaqMan probes. Copy number estimates were validated using a separate SYBR green-based quantitative PCR assay as well as a novel PCR-based breakpoint assay to detect the hybrid gene product. Field samples from 2012 to 2015 across three sites in Cambodia were tested using DNA extracted from dried blood spots and whole blood to monitor the extent of *plasmepsin 2/3* gene amplifications, as well as amplifications in the multidrug resistance transporter 1 gene (*pfmdr1*), a marker of mefloquine resistance. This study found high concordance across all methods of copy number detection. For samples derived from dried blood spots, a success rate greater than 80% was found in each assay, with more recent samples performing better. Evidence of extensive *plasmepsin 2/3* copy number amplifications was observed in Pursat (94%, 2015) (Western Cambodia) and Preah Vihear (87%, 2014) (Northern Cambodia), and lower levels in Ratanakiri (16%, 2014) (Eastern Cambodia). A shift was observed from two copies of *plasmepsin 2* in Pursat in 2013 to three copies in 2014–2015 (25% to 64%). *Pfmdr1* amplifications were absent in all samples from Preah Vihear and Ratanakiri in 2014 and absent in Pursat in 2015.

**Conclusions:**

The multiplex TaqMan assay is a robust tool for monitoring both *plasmepsin* 2/3 and *pfmdr1* copy number variations in field isolates, and the SYBR-green and breakpoint assays are useful for monitoring *plasmepsin 2/3* amplifications. This study shows increasing levels of *plasmepsin 2* copy numbers across Cambodia from 2012 to 2015 and a complete reversion of multicopy *pfmdr1* parasites to single copy parasites in all study locations.

## Background

As malaria endemic countries strive toward malaria elimination, one of the main obstacles is the continued availability of efficacious drugs. In Southeast Asia, drug resistance is wide-spread, with the most recent emergence of resistance to the current first-line treatment, artemisinin-based combination therapy (ACT) [1].

The declining efficacy of ACT in recent years can be largely attributed to rising resistance to artemisinin partner drugs, notably piperaquine. Dihydroartemisinin–piperaquine (DHA–PPQ) was introduced as the first-line treatment for malaria in 2008 in Cambodia, following therapeutic failure of artesunate–mefloquine (AS–MQ), the ACT used prior to 2008. Since 2012–2013, studies in Cambodia have shown declining efficacy to piperaquine in vitro, and subsequent increases in clinical treatment failures [2–7]. Genomic studies carried out in parallel with samples from these clinical efficacy studies have shown that there are multiple signals across the parasite genome that associate with both in vitro piperaquine resistance and clinical treatment failures [8, 9]. Specifically, a gene duplication within the *plasmepsin* multi-gene cluster on the parasite chromosome 14 and a non-synonymous SNP in a putative exonuclease gene (PF3D7_1362500) on chromosome 13, *exo*-*E415G*. Additional work also points to mutations in the chloroquine resistance transporter gene, *pfcrt* that can confer differing levels of piperaquine resistance in field and laboratory isolates [10–13].

The duplication encompasses *plasmepsin 2* and a hybrid of the *plasmepsin 1* and *3* genes that was highly correlated (adjusted hazard-ratio 16.7) with parasite recrudescence following adequate drug treatment with DHA–PPQ. This effect holds in the artemisinin resistance-associated *kelch13* propeller (*k13*) domain mutant populations [14] (adjusted hazard-ratio 5.2) suggesting an independent mechanism [8]. Members of the *plasmepsin* gene family in *P. falciparum* are involved in the haemoglobin degradation pathway, specifically in the formation of haemozoin [15]. As the parasites digest haemoglobin and release haem, toxic by-products that cause oxidative stress are formed and the conversion of intermediates to inert haemozoin crystals detoxifies the harmful by-products. The plasmepsin enzymes are redundant and other enzymes also facilitate the haemoglobin digestion pathway, including falcilysins and falcipains [16–18]. The main duplication in *plasmepsin 2/3* observed in Southeast Asia has a conserved breakpoint within the distal end of *plasmepsin 3* that includes complete duplication of the *plasmepsin 2* gene. In the same studies, an association was seen between increased copy numbers of the multidrug resistance transporter 1 gene (*pfmdr1*), a marker of mefloquine resistance, and low piperaquine IC_50_ values. It is unknown if the association between decreased piperaquine IC_50_ values and single copy *pfmdr1* is due to a drug effect, or is due to the expansion of piperaquine resistance on a mefloquine-sensitive parasite line. Some studies have shown that increased *pfmdr1* copy numbers are associated with increased susceptibility to piperaquine [19] and more recent work has suggested that *plasmepsin 2/3* amplifications and *pfmdr1* deamplifications have created a genetic background that favors *pfcrt* mutations [20]. With the observation of possible counteracting resistance mechanisms to piperaquine and mefloquine, it has been suggested to re-introduce mefloquine to areas of emerging piperaquine resistance, or to combine mefloquine into a triple ACT (TACT) with piperaquine. Both options are currently being investigated [21, 22].

Monitoring the *plasmepsin 2/3* amplification as a marker of piperaquine resistance will enable rapid determination of its frequency in populations using DHA–PPQ as well as identification of the amplification in new parasite populations. This study developed a TaqMan based quantitative PCR (qPCR) to measure the copy number of *plasmepsin 2* within the duplicated region, and have also combined it with a TaqMan assay that can detect increased copy numbers of *pfmdr1*. This single reaction multiplex qPCR can be used to efficiently monitor resistance to both piperaquine and mefloquine, which will be of particular relevance if TACT regimens are adopted in countries such as Cambodia. This study also developed a PCR-based breakpoint assay for detection of the hybrid sequence created as a result of the *plasmepsin 2/3* duplication. The breakpoint assay can be used in in conjunction with the qPCR assays or in low-resource settings where qPCR is not feasible.

## Methods

### Samples

Laboratory isolates used in qPCR validation were obtained from a clinical trial carried out in three sites in Cambodia, Pursat, Preah Vihear, and Ratanakiri, between 2012 and 2013 [2]. Blood samples from this study were taken as whole-blood venous draws following malaria diagnosis and from an initial finger prick dried blood spot (DBS). A subset of DNA samples extracted from the venous blood were whole-genome sequenced and *pfmdr1* and *plasmepsin 2* copy-numbers were called from sequence data according to Amato et al. [8]. Additional field isolates (after 2013) from clinical trials performed by Amaratunga et al. [2] at the same three sites in Cambodia as above were used to test ongoing copy-number polymorphisms (clinicaltrials.gov ID: NCT01736319).

### Quantification of *plasmepsin 2*, *plasmepsin 3*, and *pfmdr1* by real-time PCR

Primers for both *plasmepsin 2* and *plasmepsin 3* were designed using GenScript online tool (https://www.genscript.com/tools/) to match the T_m_ of the previously described *pfmdr1* TaqMan assay [23]. *Plasmepsin 2* primers (forward-5′-ATGGTGATGCAGAAGTTGGA-3′, reverse-5′-AACATCCTGCAGTTGTACATTTAAC-3′) and *plasmepsin 3* primers (forward-5′-CCACTTGTGGTAACACGAAATTA-3′; reverse-5′-TGGTTCAAGGTATTGTTTAGGTTC-3′) were selected to match the optimized reaction conditions of the *pfmdr1* primers (forward 5′-TGCATCTATAAAACGATCAGACAAA-3′, reverse 5′-TCGTGTGTTCCATGTGACTGT-3′). For the *plasmepsin* assay, the same *β*-*tubulin* single copy reference primers (forward 5′-TGATGTGCGCAAGTGATCC-3′, reverse 5′-TCCTTTGTGGACATTCTTCCTC-3′) were used as in the *pfmdr1* assay. First, the two-probe assay with either *plasmepsin 2* (5′Fam-CAGGATCTGCTAATTTATGGGTCCCA-3′BHQ-2) or *plasmepsin 3* (5′Fam-CCAACACTCGAATATCGTTCACCAA-3′BHQ-2) and *β*-*tubulin* (5′MAX-TAGCACATGCCGTTAAATATCTTCCATGTCT-3′BHQ-1) was validated first using DNA extracted from whole blood and compared with copy number estimates called from whole-genome sequence data. The *plasmepsin 2* probe set was then multiplexed with *pfmdr1* (5′ Cy5-TTTAATAACCCTGATCGAAATGGAACCTTTG-3′BHQ-2) and then tested using DNA extracted from DBS in the same set of samples. All primers and probes were ordered from Integrated DNA Technologies, Inc. Reactions were carried out in 25 μL volumes in 96 well plates (Star Labs) on a Roche LightCycler 480. For each reaction, 2× Concentrated Roche LightCycler 480 Probes Master (containing FastStart Taq DNA Polymerase), 300 nmol/L of each *plasmepsin 2* or *plasmepsin 3*, *pfmdr1*, and *β*-*tubulin* primers, 100 nmol/L of each probe, and 2–5 μL of sample DNA were used. PCR cycling conditions had an initial step of 95 °C for 10 min followed by 50 cycles of 95 °C for 15 s and 58 °C for 60 s. To calculate the fold-change, the ΔΔCt method, ΔΔCt = (Ct_TE_ − Ct_HE_) − (Ct_TC_ − Ct_HC_) was used, where T is the test gene (either *plasmepsin 2*, *plasmepsin 3*, or *pfmdr1*), H is the reference gene (*β*-*tubulin*), E is the experimental sample, and C is the control sample (for all tests 3D7 was used as the single copy control). Relative expression was calculated as 2^−ΔΔCt^.

### SYBR green validation of *plasmepsin 2* copy number using quantitative PCR

PCR primers for estimating *plasmepsin 2/3* copy number amplification were designed manually inside the *plasmepsin 2* gene (forward-5′-CTTATACTGCTTCAACATTTGATGGTATCCTTGG-3′; reverse-5′-GGTTAAGAATCCTGTATGTTTATCATGTACAGGTAAG-3′). Previously described primers for *P. falciparum lactate dehydrogenase* (*ldh*) [24], were used as the control for a single copy gene (forward-5′-AGGACAATATGGACACTCCGAT-3′; reverse-5′-TTTCAGCTATGGCTTCATCAAA-3′). Quantitative PCR (qPCR) reactions were carried out in 20 μL volumes in a 96-well plate (Bio-Rad, Hercules, CA) containing 10 μL SensiFAST SYBR No-ROX mix (2×) (Bioline Inc., Taunton, MA), 300 nM of each primer, and 2 μL genomic DNA. Reactions were performed using a CFX Connect Real-Time PCR Detection System (Bio-Rad) using the following conditions: 5 min at 95 °C, followed by 40 cycles of 10 s at 95 °C, 20 s at 58 °C, and 20 s at 60 °C. Relative copy number was calculated on the basis of the 2^−ΔΔCt^ method for relative quantification. ΔΔCt was calculated as (Ct_*ldh*_ − Ct_*pfplasmepsin2*_) − (Ct_*ldh* cal_ − *Ct*_*pfplasmepsin2* cal_), where cal is the calibration control of genomic 3D7 DNA with one copy of both *ldh* and *plasmepsin 2*. DNA from an isolate with two copies of *plasmepsin 2/3* (PH1387-C) [2] was used as an internal plate control. All samples were analysed in triplicate and each plate was replicated in triplicate.

### *Plasmepsin 2/3* duplication breakpoint PCR assay

Whole genome sequencing (WGS) data from *P. falciparum* genomic DNA collected during field studies in Cambodia was used to detect the *plasmepsin 2/3* gene amplification as previously reported [2, 8]. With the available WGS data, the breakpoint of the *plasmepsin 2/3* amplification was used to manually design PCR primers to amplify the region surrounding the breakpoint. Primers AF (forward 5′-CCACGATTTATATTGGCAAGTTGATTTAG-3′) and AR (reverse 5′-CATTTCTACTAAAATAGCTTTAGCATCATTCACG-3′) amplify a 623 base pair product surrounding the breakpoint located at the 3′ end of *plasmepsin 1*. Primers BF (forward-5′-CGTAGAATCTGCAAGTGT TTTCAAAG-3′) and BR (reverse 5′-AATGTTATAAATGCAATATAATCAAACGACATCAC-3′) amplify a 484 base pair product surrounding the breakpoint located at the 3′ end of *plasmepsin 3*. BF + AR amplify the junction between the breakpoint and produce a 497 base pair product in isolates with *plasmepsin 2/3* amplifications. These primers face opposite directions in samples without duplications and are not expected to amplify a product in single copy isolates. Both control (AF + AR; BF + BR) and duplication (BF + AR) primer sets were used with all samples and one copy isolates were only noted if the control primer sets amplified a product and duplication PCR was negative. Two or more copies were annotated as > 1 copy of *plasmepsin 2/3* only if both the control and duplication primer sets produced a product. PCR reactions contained 10 μL SapphireAmp Fast PCR Master Mix (Takara Bio USA, Mountain View, CA), 0.3 μL of each primer (10 μM stocks), 1 μL of genomic DNA up to 20 μL final volume with water. PCR conditions were: 92 °C for 2 min, followed by 30 cycles of 92 °C for 30 s, 59 °C for 30 s, 66 °C for 1.5 min, followed by a 1 min extension at 66 °C.

## Results

### Validation of copy number assays

Assays for *plasmepsin 2* and *plasmepsin 3* were designed and compared to determine if both genes could serve as markers for the entire duplication. For surveillance purposes the *plasmepsin 2* assay was multiplexed with an existing *pfmdr1* TaqMan assay. Both reference laboratory strains and culture-adapted field isolates were used to test the repeatability of each assay. The 3D7 isolate has single copies of all genes being tested while the Dd2 parasite has a duplication of the *pfmdr1* gene and the field isolates PH1265-C and PH1387-C have duplications of the *plasmepsin 2/3* complex. Two field isolates (PH1097-C and PH1310-C) have single copies of all genes and were used as baseline controls. Individual *plasmepsin* assays and the combined *plasmepsin 2*–*pfmdr1* assay showed high replicability across duplicates (Table 1).

**Table 1:** Validation of TaqMan assays with laboratory isolates

Strain	Assay	Replicates	*Plasmepsin Est. FC*		*MDR*-*1 Est. FC*	
			Avg	SD (range)	Avg	SD (range)
3D7	*plasmepsin 2*	8	1.00	0.02 (0.97–1.04)	–	–
Dd2	*plasmepsin 2*	8	1.06	0.03 (1.03–1.12)	–	–
PH1097-C	*plasmepsin 2*	6	1.06	0.02 (1.04–1.09)	–	–
PH1265-C	*plasmepsin 2*	6	1.89	0.02 (1.85–1.93)	–	–
PH1310-C	*plasmepsin 2*	6	1.06	0.03 (1.01–1.13)	–	–
PH1387-C	*plasmepsin 2*	6	1.93	0.09 (1.8–2.04)	–	–
3D7	*plasmepsin 3*	8	1.02	0.03 (0.96–1.08)	–	–
Dd2	*plasmepsin 3*	8	1.09	0.03 (1.05–1.15)	–	–
PH1097-C	*plasmepsin 3*	6	1.06	0.02 (1.03–1.09)	–	–
PH1265-C	*plasmepsin 3*	6	2.05	0.06 (1.93–2.14)	–	–
PH1310-C	*plasmepsin 3*	6	1.00	0.03 (1.02–1.09)	–	–
PH1387-C	*plasmepsin 3*	6	2.29	0.07 (2.19–2.38)	–	–
3D7	*plasmepsin 2/pfmdr1*	8	1.01	0.02 (0.97–1.04)	1.00	0.02 (0.96–1.02)
Dd2	*plasmepsin 2/pfmdr1*	8	1.10	0.04 (1.04–1.17)	1.83	0.06 (1.77–1.92)
PH1097-C	*plasmepsin 2/pfmdr1*	6	1.07	0.05 (1–1.13)	1.06	0.05 (1.01–1.11)
PH1265-C	*plasmepsin 2/pfmdr1*	6	1.80	0.03 (1.75–1.84)	0.96	0.02 (0.93–0.99)
PH1310-C	*plasmepsin 2/pfmdr1*	6	1.02	0.02 (1–1.06)	1.01	0.02 (0.98–1.05)
PH1387-C	*plasmepsin 2/pfmdr1*	6	1.89	0.05 (1.84–2)	0.93	0.03 (0.91–0.99)

**Fig. 1:**
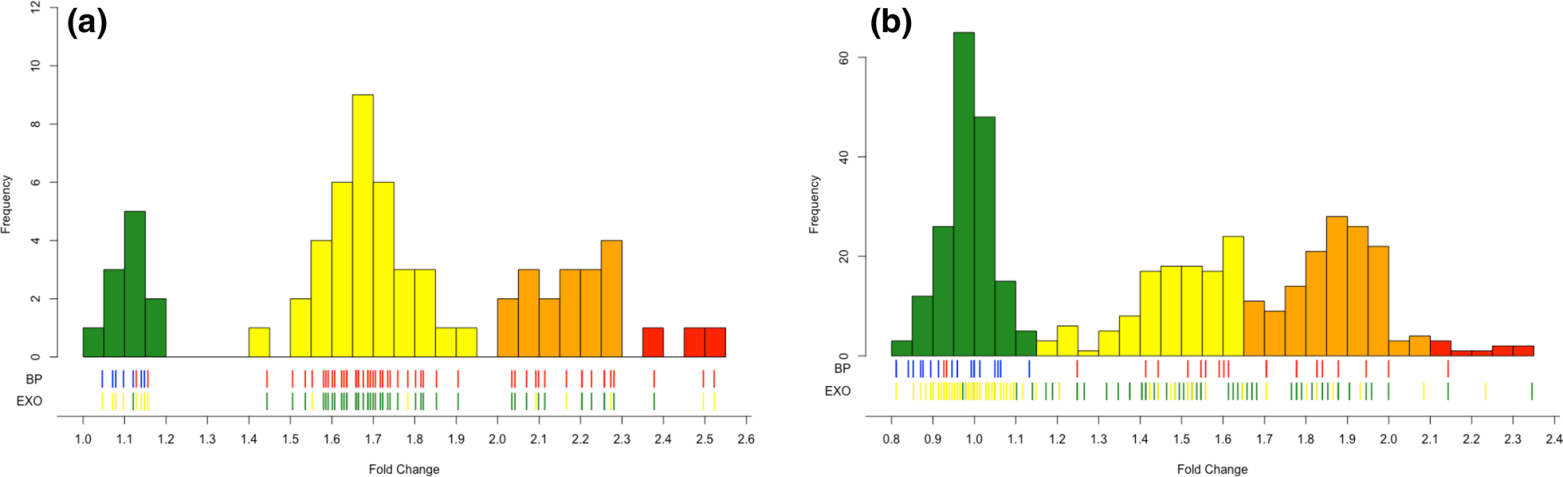
Distribution of fold-changes of samples from whole-blood (**a**) or dried blood spots (**b**) as measured by the *plasmepsin 2*–*pfmdr1* TaqMan qPCR assay. Tick marks underneath the bar graphs indicate individual sample status for the breakpoint PCR assay (BP, top line), with blue indicating no breakpoint detected and red containing the breakpoint, or *exo*-*E415G* SNP (EXO, bottom line), where yellow represents the wildtype E amino acid and green is the mutant G amino acid. Bars are coloured by their predicted *plasmepsin 2* copy number of either 1, 2, 3, or 4+ (green, yellow, orange, and red, respectively)

The TaqMan qPCR and SYBR qPCR assays were then compared using DNA extracted from whole blood for 67 patient samples. Copy number estimates from both the *plasmepsin 2* and *plasmepsin 3* assays were compared and were in 100% concordance across all samples extracted from venous blood. Fold-change values were comparable across both assays. Fifty-six of the 67 tested samples had WGS data available and had *plasmepsin 2/3* copy-number estimates available. Fifty-three of the 56 samples with copy-number estimates from WGS had the same estimate from qPCR, with the discrepancies being 1 sample called 2 copies by qPCR and 3 copies by WGS and 2 samples with 4 copies by qPCR and 3 copies by WGS. WGS estimates were used to define the relative expression boundaries between copy number estimates, and relative-expression values were lower in whole-blood extracted samples than in cultured laboratory isolates (Fig. 1a, Additional file 1). A high proportion of the *exo*-*E415G* mutation was observed among *plasmepsin 2* multi-copy parasites (Fig. 1, Additional file 1). *Exo*-*415* sequencing was determined from samples with available WGS data [2, 8].

To validate the copy number estimates, available samples were tested at the National Institutes of Health (NIH) in the Laboratory of Malaria and Vector Research (LMVR) in Bethesda, MD, USA using a separately developed SYBR-green based approach. Of the 31 samples available at both laboratories, 29 samples matched in *plasmepsin 2* copy number estimate with one sample being called 2 copies by TaqMan and 3 copies by SYBR-green, and one sample being called 3 copies by TaqMan and 2 copies by SYBR-green. All dual-tested samples that had copy number estimates from both the SYBR-green method and WGS were in 100% concordance.

### Duplication breakpoint assay validation

**Fig. 2:**
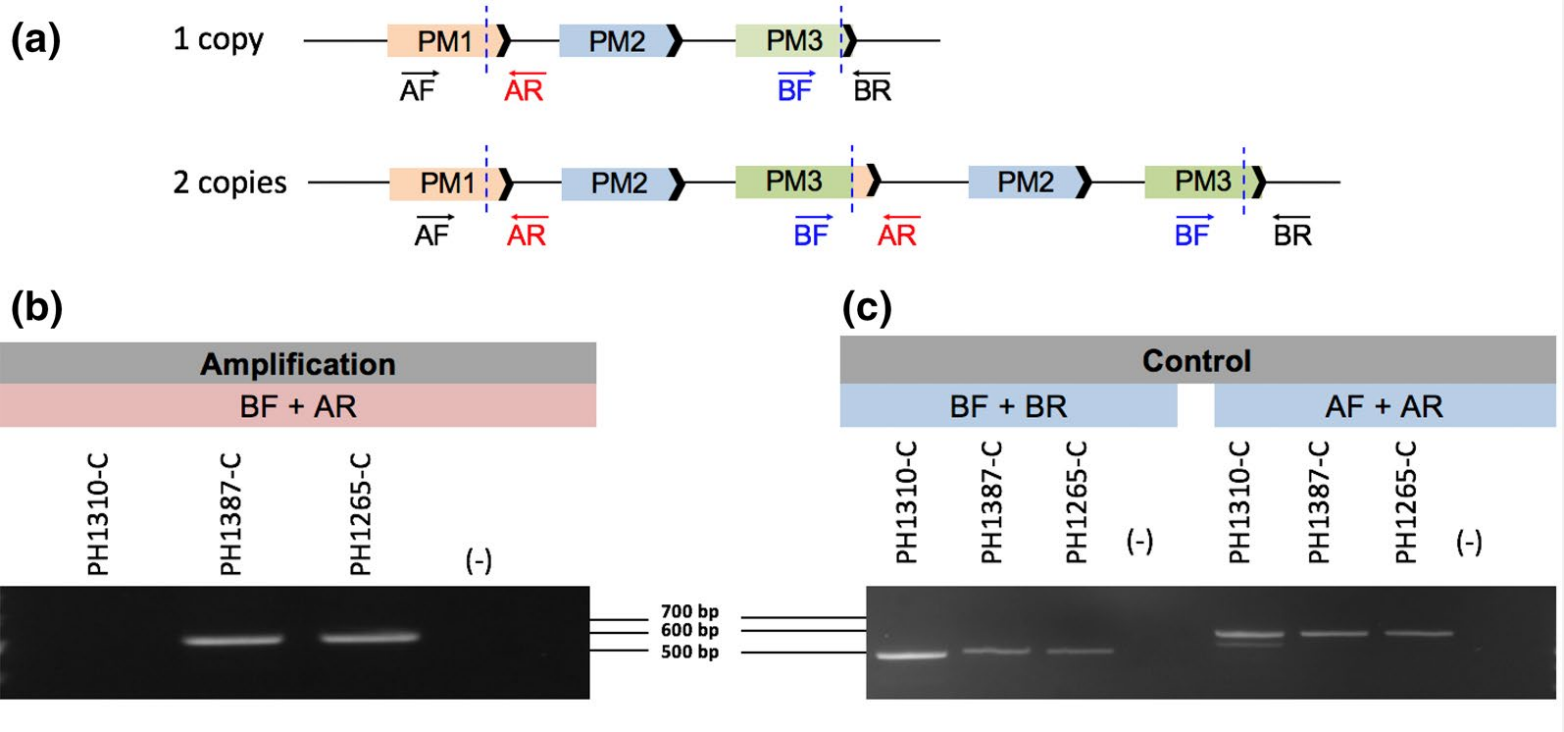
Schematic of *plasmepsin 2/3 gene* duplication. **a** Gene model depicting the *plasmepsin 2/3* breakpoint (dashed blue lines) observed in Cambodian isolates. Primer positions are labeled in the single copy (top) and multi-copy (bottom) isolates. **b** Amplification primer set BF + AR amplifies a product in an isolate with two copies (PH1387-C) and three copies (PH1265-C) of *plasmepsin 2/3*. No product is observed for the single copy (PH1310-C) isolate or in the DNA-negative control (−). **c** Control primers amplify a product in the single copy (PH1310-C) and multi-copy isolates (PH1387-C; PH1265-C). No product is observed in the DNA-negative control (−)

PCR primers that amplify the breakpoint of the *plasmepsin 2/3* amplification observed in Cambodian isolates were designed (Fig. 2a). The breakpoint assay identifies copy number amplification in isolates that contain 2 or greater copies of *plasmepsin 2* and *3* (Fig. 2b). As expected, no PCR products were observed in samples with a single copy of *plasmepsin 2/3* (Fig. 2b). Control primers confirmed that the regions surrounding the *plasmepsin 2/3* amplification were present in all isolates (Fig. 2c).

Further verification of the breakpoint assay was performed via sequence analysis of the BF + AR PCR product. Sequence data revealed the same breakpoint location as observed by WGS [8]. All Cambodian samples that were positive for *plasmepsin 2/3* amplification as detected by the breakpoint assay (> 1 copy) were in 100% concordance with qPCR and WGS data that called 2 or more copies of *plasmepsin 2*. Sequencing chromatogram review of PCRs representing 2 and 3 copy samples showed that the breakpoints for representative 2 and 3 copy samples were identical. These sequencing results combined with the identical PCR sizes for all isolates indicates that the breakpoint is identical in all Cambodian isolates tested to date.

To test the utility of using the breakpoint assay for rapid surveillance of large sample sizes for which WGS data is not yet completed or available the presence of amplification in an additional 99 samples was analysed. The results showed that 93/99 (94%) samples tested with the breakpoint PCR assay matched qPCR data for the same samples (Additional file 1). The six non-concordant samples were repeated, always producing the same results with the breakpoint detecting a copy-number increase and the qPCR single-copy. These results suggest that the breakpoint PCR assay is more sensitive than the qPCR assay for detecting minor clones containing the duplication in field isolates.

### Copy number surveillance in Cambodia

To test the feasibility of using this assay as a surveillance tool, the *plasmepsin 2*–*pfmdr1* TaqMan assay was performed on 524 samples extracted from DBS collected in drug efficacy studies from 3 field sites within Cambodia (Pursat, Preah Vihear and Ratanakiri) from 2012 to 2015 (Additional file 1). There was an 84% (success rate across all samples, but success rate improved with time since collection (2012, 68%; 2013, 72%; 2014, 94%; 2015, 100%). Assays run on samples from DBS were checked against SYBR-green results run on whole-blood extracted samples analysed at the NIH LMVR. A total of 171 samples were available for analysis using both qPCR assays and had an 88% overall concordance, with only 6 (3%) samples having a discrepancy between calling a single versus multi-copy parasite. Most differences were between calling 2 versus 3 copies in either assay. Additionally, 71 samples that were either unavailable for DBS extraction or failed TaqMan qPCR were able to be typed by the SYBR-green method, giving copy-number estimates for 509 total samples. Samples with qPCR estimates in multiple sample types (whole-blood and DBS) were used to define relative expression limits between number of copies. A drop in relative expression in samples from DBS compared to whole-blood samples was noted, but confirmation of multiple copies was performed by the breakpoint assay (Fig. 1b).

**Fig. 3:**
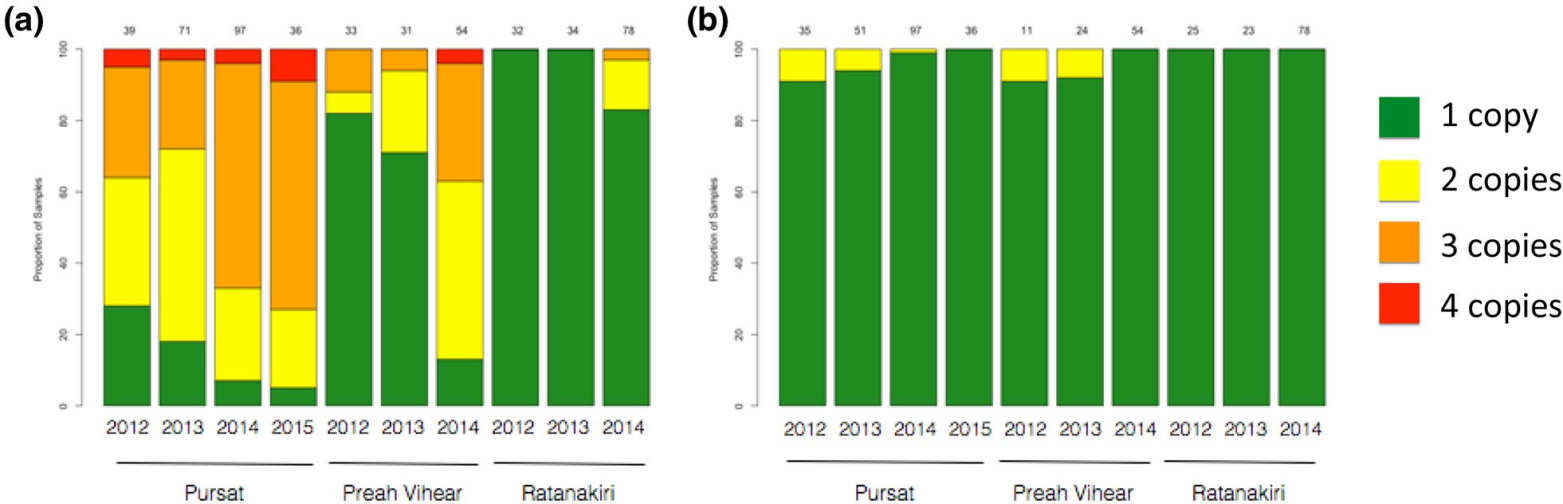
Proportion of samples by copy-number of *plasmepsin 2* (**a**) and *pfmdr1* (**b**). Bars represent proportion of samples by site and year for assays of dried-blood spot derived samples, and are coloured by number of copies detected

An increase in multi-copy *plasmepsin 2* each year was observed in both Pursat and Preah Vihear, but multi-copy parasites were not observed in Ratanakiri until 2014 when they were found in 17% of parasites analysed. An increase in parasites containing 3 copies of *plasmepsin 2* from 2012–2013 to 2014–2015 to where they are now the majority (64%) was observed. There was also a large increase in multi-copy containing parasites in Preah Vihear from 2013 (29%) to 2014 (87%) (Fig. 3a). Among all 438 samples with *pfmdr1* copy-number estimates, only 10 (2%) had multiple copies and no parasites with duplications were seen in the most recently sampled year for each site (Fig. 3b).

## Discussion

This newly developed TaqMan assay accurately determines *plasmepsin 2* and *pfmdr1* gene amplifications in samples from DBS and can be performed using standard lab equipment and a suitable qPCR machine. The specificity of TaqMan probes combined with the different absorbance spectra of labelling dyes create a system for typing the reference gene simultaneously with one or more experimental genes, making it higher throughput than SYBR-green based methods. This method shows high typeability in samples collected on dried filter paper blood spots making it ideal for surveillance of populations across wide areas. The breakpoint PCR assay also effectively determines increased *plasmepsin 2/3* copy number in field isolates and can be used in areas where qPCR is infeasible. The sensitivity of this assay enables the detection of minor clones, which proves advantageous, given the potential for polyclonal infections. However, a notable disadvantage of this method is the inability to distinguish the relative copy number (2, 3, or 4+ copies vs. > 1) in comparison with qPCR. The breakpoint assay was also designed with primers specific to the *plasmepsin 2/3* breakpoint observed in Cambodian field isolates [8]. The PCR primers used to detect the Cambodian breakpoint observed to date may not amplify a product in samples that contain different breakpoints. As previous studies have suggested [25, 26], copy number variations in the same gene can arise independently on different genetic backgrounds and result in distinct molecular breakpoints of amplification. Since qPCR methods do not rely on the location of primers in reference to an estimated breakpoint, they can be used broadly. Furthermore, the high-throughput potential of the qPCR assay to type multiple genes in the same assay while providing actual copy number estimates makes the TaqMan assay the preferred method in areas where qPCR is possible.

Since the first *Plasmodium falciparum* genome was sequenced in 2002 [27], nearly 10,000 additional genomes have been sequenced from parasites collected around the endemic world. While the first genome may not have made great strides to discover new drug or vaccine targets or point towards complicated mechanisms of disease, it was the first step in understanding the great complexity of the *Plasmodium* genomic landscape. It is this understanding that has led to great advances in determining new molecular markers of drug resistance. Historically, it has taken decades to determine a molecular cause of drug resistance, as was the case with chloroquine [28]. The availability of new methods and a catalogue of genomic variation allowed for rapid discovery and publishing of a candidate molecular marker of artemisinin resistance in 2014 [14], shortly after reports of suspected drug resistance to artemisinin in Southeast Asia were published in 2008 [29] and 2009 [30].

In 2014, the first report of DHA–PPQ treatment failures was published [7]. Because of an ever-increasing catalogue of sequenced samples and clinical and laboratory studies, a putative marker of drug resistance was published less than 2 years after the first reports of resistance [8, 9], with additional studies identifying new markers in *pfcrt* shortly after [10, 11]. Being able to extensively type variation by WGS made it possible to identify the association of increased piperaquine IC_50_ values with both SNPs and a copy-number variation. The copy-number variation at the *plasmepsin 2/3* locus showed high correlation with the phenotype and the new assays for detection will assist in monitoring its frequency in established populations, and can also monitor unaffected populations to check for new emergence or spread.

For a molecular marker to be effective in surveillance, it must be easily typed in field-derived samples. Most molecular markers of *P. falciparum* drug resistance are SNPs and can be typed by simple PCR assays [31–36], although copy-number markers exist for mefloquine [23] and antifolates [37], they are more difficult to type. Quantitative PCR makes for an easy and inexpensive method to determine copy number in samples, and unlike breakpoint assays, qPCR can determine the number of copies. It is not known if more than 2 copies of *plasmepsin 2/3* have a phenotypic effect but the increase in samples with 3 or more copies suggest some sort of selective mechanism. It is possible that a third copy of *plasmepsin 2/3* prevents a loss of resistance if the duplication is unstable and an extra copy is lost, therefore the third copy would act as a “buffer.”

Recent studies in *Plasmodium* have provided insight into the functional role of the *plasmepsins* in response to piperaquine pressure. Loesbanluechai et al. [38] showed that overexpression of *plasmepsin 2* and *plasmepsin 3* in the 3D7 parasite background did not change parasite susceptibility to piperaquine, artemisinin, or chloroquine. Other studies have suggested that *plasmepsin 2* and *plasmepsin 3* knockouts in the same 3D7 background showed decreased piperaquine survival as measured by IC_50_ values [39]. More recent studies by Silva et al. [20] using transgenic parasites with copy number variations in *plasmepsin 2/*3 showed that *pfmdr1* deamplified in the presence of the *plasmepsin 2/3* amplifications. Thus, additional functional work is needed to fully understand the relevance of the *plasmepsin 2/3* gene amplification in conferring any survival or fitness advantages in response to PPQ pressure.

The importance of examining the role of the *plasmepsin 2/3* amplification in piperaquine resistance has been further demonstrated by studies that have detected *plasmepsin 2* amplifications in several African countries. Inoue et al., Gupta et al., and Leroy et al. [40–42] have reported *plasmepsin 2* duplications in Mozambique, Mali, Gabon, Burkina Faso, and Uganda where DHA–PPQ is currently used and remains effective. It is possible that *plasmepsin 2/*3 amplifications will be found in other regions where piperaquine has been used as a partner drug and thus continued surveillance of *plasmepsin 2/3* copy numbers will prove necessary.

Mefloquine is now being re-introduced as a partner drug for artemisinin, either as a dual or triple combination therapy, in areas where DHA–PPQ is no longer effective [21]. It was removed as the first-line treatment following widespread resistance via copy number amplifications of the *pfmdr1* gene. Since mefloquine’s removal as a nationally-recommended treatment the levels of *pfmdr1* multi-copy number parasites has decreased and now is no longer detected in the samples from three distant sites in Cambodia (Fig. 3, Additional file 1). It has been suggested that a counter-acting mechanism of action has selected against multiple copies of *pfmdr1* in parasites subjected to piperaquine. This is feasible but will require confirmation, while another possibility is that the *plasmepsin 2/3* multi-copy parasites emerged on a *pfmdr1* single copy parasite background and it is this lineage(s) that have expanded.

In order to effectively monitor the spread of anti-malarial drug resistance, it is imperative to have robust, high-throughput methods for detecting genetic markers of resistance. Accurate and timely surveillance of drug resistance markers aids in maintaining and prolonging the efficacy of the limited selection of anti-malarial drugs available. The TaqMan qPCR, SYBR qPCR, and breakpoint assays described here provide a way of typing *plasmepsin 2/3* amplification, that can be readily combined with TaqMan typing of *pfmdr1* amplifications, to monitor genetic markers of mefloquine and piperaquine resistance in areas where these important ACT partner drugs are used as frontline treatment for malaria.

## Conclusions

The emergence of DHA–PPQ resistance greatly threatens the efficacy of the remaining ACTs worldwide. With the availability of *plasmepsin 2/3* amplifications as molecular markers of piperaquine resistance, it is necessary to have robust assays that can be used to monitor the presence and frequency of these markers in contemporary parasite isolates across endemic regions. This study has developed a multiplex TaqMan qPCR assay that measures both *plasmepsin* 2 and *pfmdr1* copy number, a marker of mefloquine resistance, and a SYBR-green qPCR assay for monitoring *plasmepsin 2* copy number in areas where multiplex qPCR is not possible. A PCR-based breakpoint assay was also developed to detect the presence of the *plasmepsin 2/3* amplification breakpoint reported in Cambodia. Using these methods, this study shows increasing levels of *plasmepsin 2* copy numbers across Cambodia from 2012 to 2015 and a reversion of multicopy *pfmdr1* parasites to single copy isolates in 2014–2015. The tools developed by this study will enable continued surveillance of *plasmepsin 2–pfmdr1* amplifications in regions where piperaquine and mefloquine are used as ACT partner drugs.

## Supplementary information

**Additional file 1.** The additional data table includes *plasmepsin 2/3* copy number estimates using the various methods tested in this manuscript (SYBR qPCR, TaqMan qPCR, and breakpoint PCR) compared to whole genome sequencing (WGS) data, when available. Parasite density is listed at time 0 (upon enrollment) when the samples were collected as dried blood spots (DBS) or whole blood (WB), from which gDNA was later extracted.

## Data Availability

Data sharing is not applicable to this article as no datasets were generated or analysed during the current study.

## Abbreviations

ACT: Artemisinin-based combination therapy
DBS: Dried blood spot
DHA–PPQ: Dihydroartemisinin–piperaquine
IC_50_: 50% inhibitory concentration
LMVR: Laboratory of Malaria and Vector Research
NIH: National Institutes of Health
qPCR: Quantitative polymerase chain reaction
WGS: Whole-genome sequencing
